# Design of an Optimal Preview Controller for Linear Discrete-Time Descriptor Noncausal Multirate Systems

**DOI:** 10.1155/2014/965915

**Published:** 2014-01-23

**Authors:** Mengjuan Cao, Fucheng Liao

**Affiliations:** ^1^School of Mathematics and Physics, University of Science and Technology Beijing, Beijing 100083, China; ^2^School of Automation and Electrical Engineering, University of Science and Technology Beijing, Beijing 100083, China

## Abstract

The linear discrete-time descriptor noncausal multirate system is considered for the presentation of a new design approach for optimal preview control. First, according to the characteristics of causal controllability and causal observability, the descriptor noncausal system is constructed into a descriptor causal closed-loop system. Second, by using the characteristics of the causal system and elementary transformation, the descriptor causal closed-loop system is transformed into a normal system. Then, taking advantage of the discrete lifting technique, the normal multirate system is converted to a single-rate system. By making use of the standard preview control method, we construct the descriptor augmented error system. The quadratic performance index for the multirate system is given, which can be changed into one for the single-rate system. In addition, a new single-rate system is obtained, the optimal control law of which is given. Returning to the original system, the optimal preview controller for linear discrete-time descriptor noncausal multirate systems is derived. The stabilizability and detectability of the lifted single-rate system are discussed in detail. The optimal preview control design techniques are illustrated by simulation results for a simple example.

## 1. Introduction

Descriptor system theory has obtained many excellent results in the control areas; the main scholarly reports can be seen in [1, 2]. In recent years, the literature [3] considered the optimal fusion problem for the state estimation of discrete-time stochastic singular systems with multiple sensors and correlated measurement noise and obtained the optimal full-order filters and smoothers for the original system. The literature [4] proposed a novel suboptimal control method for a class of nonlinear singularly perturbed systems based on adaptive dynamic programming; the literature [5] discussed finite-time robust dissipative control for a class of descriptor systems, and the control system was effectively confined within the desired state-space ellipsoid. The literature [6] provided a necessary and sufficient condition to guarantee admissibility for positive continuous-time descriptor systems. Notably, the literature [7] combined descriptor system theory with preview control theory and successfully obtained the optimal preview controller with preview action for the linear discrete-time descriptor causal system; the literature [8] derived the optimal preview controller for discrete-time descriptor causal systems in a multirate setting. The literature [9] obtained the optimal preview controller with preview feedforward compensation for linear discrete-time descriptor systems with state delay. In addition, linear quadratic optimal regulator theory for the continuous and discrete descriptor system tends to be complete as discussed in [10–12].

In recent years, the multirate digital control system has also obtained many new results as discussed in [13–16]. The characteristics of multirate systems are as follows. First of all, the systems are multiinput and multioutput systems. Second, the sampler and retainer of input channels and output channels have different sampling periods as discussed by Xiao [13]. For such systems, if the designed regulator satisfies appropriate multirate characteristics, it should have a better performance than that of the single-rate regulator.

The previous multirate systems have been basically studied for normal systems; however, this paper successfully constructs the optimal preview controller on the basis of the literature [8] for linear discrete-time descriptor noncausal multirate systems. The effectiveness of the proposed method is shown by simulation.

## 2. Description of the Problem and Basic Assumptions

Consider the regular linear discrete-time descriptor noncausal system described by

(1) $$\begin{array}{c} Ex\left(k+1\right)=Ax\left(k\right)+Bu\left(k\right),\\ y\left(k\right)=Cx\left(k\right), \end{array}$$

where *x*(*k*) ∈ *R* ^*n*^, *u*(*k*) ∈ *R* ^*r*^, and *y*(*k*) ∈ *R* ^*m*^ are its state, control input, and measure output, respectively; *E*, *A* ∈ *R* ^*n*×*n*^, *B* ∈ *R* ^*n*×*r*^, and *C* ∈ *R* ^*m*×*n*^ are constant matrices; here, *E* is a singular matrix with rank⁡(*E*) = *q* < *n*.

As [8], we need to make the following basic assumptions:

 Assumption 1 (A1): system (1) is stabilizable.
 Assumption 2 (A2): system (1) is detectable.
 Assumption 3 (A3): the system (1) is both causally controllable and causally observable.
 Assumption 4 (A4): the state vector *x*(*k*) and output vector *y*(*k*) can only be measured at *k* = *iN*  (*i* = 0,1, 2,…), where *N* is a positive integer.
 Assumption 5 (A5): the preview length of the reference signal *R*(*k*) is *M*_*R*_; that is, at each time *k*, the *M*_*R*_ future values *R*(*k* + 1), *R*(*k* + 2),…, *R*(*k* + *M*_*R*_), and the present and past values of the reference signal are available where *M*_*R*_ = *SN* and *S* is a nonnegative integer.

The future values of the desired signal are assumed not to change beyond the *k* + *M*_*R*_; namely

(2) $$\begin{array}{c} R\left(k+j\right)=R\left(k+M_{R}\right),\;j=M_{R}+1,\;\;M_{R}+2,\ldots . \end{array}$$

**Remark 1**

(A1)–(A3) and (A5) are the basic assumptions, and (A4) makes the system multirate.

By (A3), there must exist a static output feedback

(3) u(k)=My(k)+v(k)=MCx(k)+v(k)=Kx(k)+v(k),

where *K* = *MC*, *v*(*k*) ∈ *R* ^*r*^, and *M* ∈ *R* ^*r*×*m*^ such that the closed-loop system

(4) $$\begin{array}{c} Ex\left(k+1\right)=\left(A+BK\right)x\left(k\right)+Bv\left(k\right) \end{array}$$

is causal as discussed by [2]; that is,

(5) $$\begin{array}{c} \deg\left\{\det\left[sE-\left(A+BK\right)\right]\right\}=\mathrm{rank}\left(E\right). \end{array}$$

Obviously, taking advantage of the characteristic that any matrix can be transformed to a canonical form by elementary transformation, there always exist nonsingular matrices *Q*_1_, *P*_1_, such that $QEP=\left[\begin{array}{cc} I_{q} & 0\\ 0 & 0 \end{array}\right]$. Denote

(6) $$\begin{array}{c} x\left(k\right)=P_{1}\left[\begin{array}{c} x_{1}\left(k\right)\\ x_{2}\left(k\right) \end{array}\right],\;\;Q_{1}\left(A+BK\right)P_{1}=\left[\begin{array}{cc} A_{11} & A_{12}\\ A_{21} & A_{22} \end{array}\right],\\ Q_{1}B=\left[\begin{array}{c} B_{1}\\ B_{2} \end{array}\right],\;\;CP_{1}=\left[\begin{array}{cc} C_{1} & C_{2} \end{array}\right], \end{array}$$

where *x*_1_(*k*) ∈ *R* ^*q*^ and *x*_2_(*k*) ∈ *R* ^*n*−*q*^.

As [8], the system (4) is restricted equivalent to

(7) $$\begin{array}{c} x_{1}\left(k+1\right)=A_{11}x_{1}\left(k\right)+A_{12}x_{2}\left(k\right)+B_{1}v\left(k\right),\\ 0=A_{21}x_{1}\left(k\right)+A_{22}x_{2}\left(k\right)+B_{2}v\left(k\right),\\ y\left(k\right)=C_{1}x_{1}\left(k\right)+C_{2}x_{2}\left(k\right). \end{array}$$

Because elementary transformation does not change the causality of the system, the system (7) is also a causal system. As a result, matrix *A*_22_ is nonsingular as discussed by [2]. Then the optimal preview problem for the descriptor noncausal system is transformed into the one for the descriptor causal system.

As [8], let the error signal

(8) $$\begin{array}{c} e\left(k\right)=y\left(k\right)-R\left(k\right). \end{array}$$

We want to get

(9) $$\begin{array}{c} \underset{k\to \infty }{\lim}e\left(k\right)=\underset{k\to \infty }{\lim}\left[y\left(k\right)-R\left(k\right)\right]=0. \end{array}$$

The quadratic performance index function for the system (1) is defined as

(10) $$\begin{array}{c} J=\sum_{k=1}^{\infty }\left[e^{T}\left(k\right)Q_{e}e\left(k\right)+\Delta u^{T}\left(k\right)H_{u}\Delta u\left(k\right)\right], \end{array}$$

where the weight matrices satisfy *Q*_*e*_ > 0 and *H*_*u*_ > 0. Δ is the first-order forward difference operator; that is, Δ*u*(*k*) = *u*(*k* + 1) − *u*(*k*).

In order to smooth the conduct of the study, we will also make the following assumptions.

Assumption 6 (A6): the matrix

(11) $$\begin{array}{c} \left[\begin{array}{ccccc} {\overset{-}{K}}_{2} & 0 & 0 & \cdots & 0\\ {\overset{-}{K}}_{1}{\overset{-}{B}}_{1} & {\overset{-}{K}}_{2} & 0 & \cdots & 0\\ {\overset{-}{K}}_{1}{\overset{-}{A}}_{1}{\overset{-}{B}}_{1} & {\overset{-}{K}}_{1}{\overset{-}{B}}_{1} & {\overset{-}{K}}_{2} & \cdots & 0\\ \vdots & \vdots & \vdots & \ddots & 0\\ {\overset{-}{K}}_{1}{\overset{-}{A}}_{1}^{N-2}{\overset{-}{B}}_{1} & {\overset{-}{K}}_{1}{\overset{-}{A}}_{1}^{N-3}{\overset{-}{B}}_{1} & {\overset{-}{K}}_{1}{\overset{-}{A}}_{1}^{N-4}{\overset{-}{B}}_{1} & \cdots & {\overset{-}{K}}_{2} \end{array}\right] \end{array}$$

is nonsingular, where the meaning of the various symbols is given in the following discussion.

Assumption 7(A7): the matrix

(12) $$\begin{array}{c} \Psi =\left[\begin{array}{cccccc} {\overset{-}{A}}_{1}^{N}-I & {\overset{-}{A}}_{1}^{N-1}{\overset{-}{B}}_{1} & {\overset{-}{A}}_{1}^{N-2}{\overset{-}{B}}_{1} & \cdots & {\overset{-}{A}}_{1}{\overset{-}{B}}_{1} & {\overset{-}{B}}_{1}\\ {\overset{-}{C}}_{1} & {\overset{-}{C}}_{2} & 0 & \cdots & \cdots & 0\\ {\overset{-}{C}}_{1}{\overset{-}{A}}_{1} & {\overset{-}{C}}_{1}{\overset{-}{B}}_{1} & {\overset{-}{C}}_{2} & \ddots & & \vdots \\ \vdots & \vdots & & \ddots & \ddots & \vdots \\ {\overset{-}{C}}_{1}{\overset{-}{A}}_{1}^{N-2} & {\overset{-}{C}}_{1}{\overset{-}{A}}_{1}^{N-3}{\overset{-}{B}}_{1} & {\overset{-}{C}}_{1}{\overset{-}{A}}_{1}^{N-4}{\overset{-}{B}}_{1} & \cdots & {\overset{-}{C}}_{2} & 0\\ {\overset{-}{C}}_{1}{\overset{-}{A}}_{1}^{N-1} & {\overset{-}{C}}_{1}{\overset{-}{A}}_{1}^{N-2}{\overset{-}{B}}_{1} & {\overset{-}{C}}_{1}{\overset{-}{A}}_{1}^{N-3}{\overset{-}{B}}_{1} & \cdots & {\overset{-}{C}}_{1}{\overset{-}{B}}_{1} & {\overset{-}{C}}_{2} \end{array}\right] \end{array}$$

is of full row rank, where the meaning of the various symbols is given in the following discussion.

## 3. The Derivation of the Single-Rate System

The system (7) is a multirate system according to the above discussion. We adopt the discrete lifting technique to convert (7) to a single-rate system.

By the obtained results in [8], (7) can be lifted as

(13) $$\begin{array}{c} {\tilde{x}}_{1}\left(i+1\right)={\overset{-}{A}}_{1}^{N}{\tilde{x}}_{1}\left(i\right)+{\tilde{B}}_{1}\tilde{v}\left(i\right),\\ \tilde{y}\left(i\right)={\tilde{C}}_{1}{\tilde{x}}_{1}\left(i\right)+{\tilde{C}}_{2}\tilde{v}\left(i\right), \end{array}$$

where

(14) $$\begin{array}{c} {\overset{-}{A}}_{1}=A_{11}-A_{12}A_{22}^{-1}A_{21},\\ {\overset{-}{B}}_{1}=B_{1}-A_{12}A_{22}^{-1}B_{2},\;\;{\tilde{x}}_{1}\left(i\right)=x_{1}\left(iN\right)\in R^{q},\\ {\tilde{x}}_{2}\left(i\right)=x_{2}\left(iN\right)\in R^{n-q},\;\;\tilde{v}\left(i\right)=\left[\begin{array}{c} v\left(iN\right)\\ \vdots \\ v\left(iN+N-2\right)\\ v\left(iN+N-1\right) \end{array}\right],\\ {\tilde{B}}_{1}=\left[\begin{array}{cccc} {\overset{-}{A}}_{1}^{N-1}{\overset{-}{B}}_{1} & \cdots & {\overset{-}{A}}_{1}{\overset{-}{B}}_{1} & {\overset{-}{B}}_{1} \end{array}\right],\\ {\overset{-}{C}}_{1}=C_{1}-C_{2}A_{22}^{-1}A_{21},\\ {\overset{-}{C}}_{2}=-C_{2}A_{22}^{-1}B_{2},\;\;\tilde{y}\left(i\right)=\left[\begin{array}{c} y\left(iN\right)\\ y\left(iN+1\right)\\ \vdots \\ y\left(iN+N-2\right)\\ y\left(iN+N-1\right) \end{array}\right],\\ {\tilde{C}}_{1}=\left[\begin{array}{c} {\overset{-}{C}}_{1}\\ {\overset{-}{C}}_{1}{\overset{-}{A}}_{1}\\ \vdots \\ {\overset{-}{C}}_{1}{\overset{-}{A}}_{1}^{N-2}\\ {\overset{-}{C}}_{1}{\overset{-}{A}}_{1}^{N-1} \end{array}\right],\\ {\tilde{C}}_{2}=\left[\begin{array}{ccccc} {\overset{-}{C}}_{2} & 0 & \cdots & 0 & 0\\ {\overset{-}{C}}_{1}{\overset{-}{B}}_{1} & {\overset{-}{C}}_{2} & \cdots & 0 & 0\\ \vdots & \vdots & \ddots & \vdots & \vdots \\ {\overset{-}{C}}_{1}{\overset{-}{A}}_{1}^{N-3}{\overset{-}{B}}_{1} & {\overset{-}{C}}_{1}{\overset{-}{A}}_{1}^{N-4}{\overset{-}{B}}_{1} & \cdots & {\overset{-}{C}}_{2} & 0\\ {\overset{-}{C}}_{1}{\overset{-}{A}}_{1}^{N-2}{\overset{-}{B}}_{1} & {\overset{-}{C}}_{1}{\overset{-}{A}}_{1}^{N-3}{\overset{-}{B}}_{1} & \cdots & {\overset{-}{C}}_{1}{\overset{-}{B}}_{1} & {\overset{-}{C}}_{2} \end{array}\right]. \end{array}$$

In order to design the optimal preview controller for linear discrete-time descriptor noncausal multirate systems (1), continue to lift the static output feedback (3).

First, denoting $KP_{1}=\left[\begin{array}{cc} K_{1} & K_{2} \end{array}\right]$, where *K*_1_ ∈ *R* ^*r*×*q*^ and *K*_2_ ∈ *R* ^*r*×(*n*−*q*)^, we have

(15) u(k)=KP1[x1(k)x2(k)]+v(k)=[K1K2][x1(k)x2(k)]+v(k)=K1x1(k)+K2x2(k)+v(k).

We know that matrix *A*_22_ is nonsingular. Then we can derive

(16) $$\begin{array}{c} x_{2}\left(k\right)=-A_{22}^{-1}A_{21}x_{1}\left(k\right)-A_{22}^{-1}B_{2}v\left(k\right), \end{array}$$

from the second equation of (7).

By (16), (15) will become

(17) u(k)=K1x1(k)+K2(−A22−1A21x1(k)−A22−1B2v(k)) +v(k)=(K1−K2A22−1A21)x1(k) +(I−K2A22−1B2)v(k)=K−1x1(k)+K−2v(k),

where

(18) $$\begin{array}{c} {\overset{-}{K}}_{1}=K_{1}-K_{2}A_{22}^{-1}A_{21},\;\;{\overset{-}{K}}_{2}=I-K_{2}A_{22}^{-1}B_{2}. \end{array}$$

Substituting (16) into the first equation in (7), we get

(19) $$\begin{array}{c} x_{1}\left(k+1\right)={\overset{-}{A}}_{1}x_{1}\left(k\right)+{\overset{-}{B}}_{1}v\left(k\right). \end{array}$$

Using (19) repeatedly, (17) will become

(20) u(iN)=K−1x1(iN)+K−2v(iN),u(iN+1)=K−1x1(iN+1)+K−2v(iN+1)=K−1(A−1x1(iN)+B−1v(iN))+K−2v(iN+1)=K−1A−1x1(iN)+K−1B−1v(iN)+K−2v(iN+1),u(iN+2)=K−1A−1x1(iN+1)+K−1B−1v(iN+1)+K−2v(iN+2)=K−1A−1(A−1x1(iN)+B−1v(iN))+K−1B−1v(iN+1)+K−2v(iN+2)=K−1A−12x1(iN)+K−1A−1B−1v(iN)+K−1B−1v(iN+1)+K−2v(iN+2),          ⋮u(iN+N−1)=K−1A−1N−1x1(iN)+K−1A−1N−2B−1v(iN)+⋯+K−1B−1v(iN+N−2)+K−2v(iN+N−1).

The above equations may be represented in the matrix form:

(21) $$\begin{array}{c} \tilde{u}\left(i\right)={\tilde{K}}_{1}{\tilde{x}}_{1}\left(i\right)+{\tilde{K}}_{2}\tilde{v}\left(i\right), \end{array}$$

where

(22) $$\begin{array}{c} {\tilde{K}}_{1}=\left[\begin{array}{c} {\overset{-}{K}}_{1}\\ {\overset{-}{K}}_{1}{\overset{-}{A}}_{1}\\ {\overset{-}{K}}_{1}{\overset{-}{A}}_{1}^{2}\\ \vdots \\ {\overset{-}{K}}_{1}{\overset{-}{A}}_{1}^{N-1} \end{array}\right],\\ {\tilde{K}}_{2}=\left[\begin{array}{ccccc} {\overset{-}{K}}_{2} & 0 & 0 & \cdots & 0\\ {\overset{-}{K}}_{1}{\overset{-}{B}}_{1} & {\overset{-}{K}}_{2} & 0 & \cdots & 0\\ {\overset{-}{K}}_{1}{\overset{-}{A}}_{1}{\overset{-}{B}}_{1} & {\overset{-}{K}}_{1}{\overset{-}{B}}_{1} & {\overset{-}{K}}_{2} & \cdots & 0\\ \vdots & \vdots & \vdots & \ddots & 0\\ {\overset{-}{K}}_{1}{\overset{-}{A}}_{1}^{N-2}{\overset{-}{B}}_{1} & {\overset{-}{K}}_{1}{\overset{-}{A}}_{1}^{N-3}{\overset{-}{B}}_{1} & {\overset{-}{K}}_{1}{\overset{-}{A}}_{1}^{N-4}{\overset{-}{B}}_{1} & \cdots & {\overset{-}{K}}_{2} \end{array}\right],\\ \tilde{u}\left(i\right)=\left[\begin{array}{c} u\left(iN\right)\\ u\left(iN+1\right)\\ u\left(iN+2\right)\\ \vdots \\ u\left(iN+N-1\right) \end{array}\right]. \end{array}$$

**Remark 2**

${\tilde{K}}_{2}$ is exactly the matrix in (A6).

## 4. Construction of the Descriptor Augmented Error System

As [8], we take advantage of the first-order forward difference operator Δ:

(23) $$\begin{array}{c} \Delta {\tilde{x}}_{1}\left(i\right)={\tilde{x}}_{1}\left(i+1\right)-{\tilde{x}}_{1}\left(i\right). \end{array}$$

Construct the vector

(24) $$\begin{array}{c} \tilde{R}\left(i\right)=\left[\begin{array}{c} R\left(iN\right)\\ R\left(iN+1\right)\\ \vdots \\ R\left(iN+N-2\right)\\ R\left(iN+N-1\right) \end{array}\right]. \end{array}$$

Then we can obtain the error vector

(25) e~(i)=y~(i)−R~(i)=[y(iN)y(iN+1)⋮y(iN+N−2)y(iN+N−1)]−[R(iN)R(iN+1)⋮R(iN+N−2)R(iN+N−1)]=[e(iN)e(iN+1)⋮e(iN+N−2)e(iN+N−1)].

By the second equation in (13), we derive

(26) $$\begin{array}{c} \tilde{e}\left(i\right)={\tilde{C}}_{1}{\tilde{x}}_{1}\left(i\right)-\tilde{R}\left(i\right)+{\tilde{C}}_{2}\tilde{v}\left(i\right). \end{array}$$

Using Δ on both sides of (26) and noticing $\Delta \tilde{e}(i)=\tilde{e}(i+1)-\tilde{e}(i)$, we obtain

(27) $$\begin{array}{c} \tilde{e}\left(i+1\right)=\tilde{e}\left(i\right)+{\tilde{C}}_{1}\Delta {\tilde{x}}_{1}\left(i\right)-\Delta \tilde{R}\left(i\right)+{\tilde{C}}_{2}\Delta \tilde{v}\left(i\right). \end{array}$$

Using Δ on both sides of the first equation of (13), we can derive

(28) $$\begin{array}{c} \Delta {\tilde{x}}_{1}\left(i+1\right)={\overset{-}{A}}_{1}^{N}\Delta {\tilde{x}}_{1}\left(i\right)+{\tilde{B}}_{1}\Delta \tilde{v}\left(i\right). \end{array}$$

Combine (27) and (28) to produce

(29) [e~(i+1)Δx~1(i+1)]=[IC~10A−1N][e~(i)Δx~1(i)] +[−ImN0]ΔR~(i)+[C~2B~1]Δv~(i).

Contrasting (1), the observed vector can be taken as $\tilde{e}\left(i\right)$. Letting

(30) $$\begin{array}{c} X_{0}\left(i\right)=\left[\begin{array}{c} \tilde{e}\left(i\right)\\ \Delta {\tilde{x}}_{1}\left(i\right) \end{array}\right],\;\;\Phi =\left[\begin{array}{cc} I & {\tilde{C}}_{1}\\ 0 & {\overset{-}{A}}_{1}^{N} \end{array}\right],\\ G=\left[\begin{array}{c} {\tilde{C}}_{2}\\ {\tilde{B}}_{1} \end{array}\right],\;\;G_{R}=\left[\begin{array}{c} -I_{mN}\\ 0 \end{array}\right],\;\;C_{0}=\left[\begin{array}{cc} I & 0 \end{array}\right]. \end{array}$$

As [8], we have

(31) $$\begin{array}{c} X_{0}\left(i+1\right)=\Phi X_{0}\left(i\right)+G\Delta \tilde{v}\left(i\right)+G_{R}\Delta \tilde{R}\left(i\right),\\ \tilde{e}\left(i\right)=C_{0}X_{0}\left(i\right). \end{array}$$

Equation (31) is the error system, which is a normal system. For (31), the previewed desired signal is $\Delta \tilde{R}(i)$; that is, at each time *i*, $\Delta \tilde{R}(i),\Delta \tilde{R}(i+1),\ldots ,\Delta \tilde{R}(i+S-1)$ are available, and

(32) $$\begin{array}{c} \Delta \tilde{R}\left(i+l\right)=0\;\left(l=S,S+1,\ldots \right). \end{array}$$

Then we continue to construct the descriptor augmented error system and denote

(33) $$\begin{array}{c} X_{R}\left(i\right)=\left[\begin{array}{c} \Delta \tilde{R}\left(i\right)\\ \Delta \tilde{R}\left(i+1\right)\\ \vdots \\ \Delta \tilde{R}\left(i+S-1\right) \end{array}\right],\\ A_{R}=\left[\begin{array}{ccccc} 0 & I_{mN} & 0 & \cdots & 0\\ \vdots & \ddots & \ddots & \ddots & \vdots \\ \vdots & & \ddots & \ddots & 0\\ \vdots & & & \ddots & I_{mN}\\ 0 & \cdots & \cdots & \cdots & 0 \end{array}\right], \end{array}$$

where *A*_*R*_ is a *mNS* × *mNS* matrix; notice the identity *X*_*R*_(*i* + 1) = *A*_*R*_ *X*_*R*_(*i*). Using the identity and (31), we obtain

(34) $$\begin{array}{c} X_{R0}\left(i+1\right)=\Phi_{R0}X_{R0}\left(i\right)+G_{R0}\Delta \tilde{v}\left(i\right),\\ \tilde{e}\left(i\right)=C_{R0}X_{R0}\left(i\right). \end{array}$$

This is the constructed descriptor augmented error system. The dimension of the system (34) is *mNS* + *mN* + *q*, and

(35) $$\begin{array}{c} X_{R0}\left(i\right)=\left[\begin{array}{c} X_{R}\left(i\right)\\ X_{0}\left(i\right) \end{array}\right],\;\;\Phi_{R0}=\left[\begin{array}{cc} A_{R} & 0\\ G_{PR} & \Phi \end{array}\right],\;\;G_{R0}=\left[\begin{array}{c} 0\\ G \end{array}\right],\\ G_{PR}=\left[\begin{array}{cccc} G_{R} & 0 & \cdots & 0 \end{array}\right],\;\;C_{R0}=\left[\begin{array}{cc} 0 & C_{0} \end{array}\right]. \end{array}$$

## 5. Design of an Optimal Regulator for Descriptor Augmented Error Systems

As [8], we convert the performance index (10) as follows

(36) J=∑k=1∞[eT(k)Qee(k)+ΔuT(k)HuΔu(k)]=∑i=1∞∑j=0N−1[eT(iN+j)Qee(iN+j)      +ΔuT(iN+j)HuΔu(iN+j)]=∑i=1∞[e~T(i)Q−e~(i)+Δu~T(i)HΔu~(i)],

where

(37) $$\begin{array}{c} \overset{-}{Q}=\mathrm{diag}\underset{N}{\underset{︸}{\left(\begin{array}{cccc} Q_{e} & Q_{e} & \cdots & Q_{e} \end{array}\right)}}>0,\\ H=\mathrm{diag}\underset{N}{\underset{︸}{\left(\begin{array}{cccc} H_{u} & H_{u} & \cdots & H_{u} \end{array}\right)}}>0,\\ \;\;\overset{-}{Q}\in R^{Nm\times Nm},\;\;H\in R^{Nr\times Nr}. \end{array}$$

By (A6), ${{\tilde{K}}_{2}}^{T}H{\tilde{K}}_{2}>0$. Then, adopting the first-order forward difference operator on both sides of (21), the performance index (36) can be written as

(38) J=∑i=1∞[e~T(i)Q−e~(i)+Δu~T(i)HΔu~(i)]=∑i=1∞[e~T(i)Q−e~(i)+(K~1Δx~1(i)+K~2Δv~(i))T   ×H(K~1Δx~1(i)+K~2Δv~(i))]=∑i=1∞[e~T(i)Q−e~(i)+Δv~T(i)K~2THK~2Δv~(i)   +2Δv~T(i)K~2THK~1Δx~1(i)+Δx~1T(i)K~1THK~1Δx~1(i)]=∑i=1∞[e~T(i)Q−e~(i)+Δv~T(i)K~2THK~2Δv~(i)   +2Δv~T(i)K~2THK~1Δx~1(i)   +Δx~1T(i)K~1THK~2[K~2THK~2]−1K~2THK~1Δx~1(i)   +Δx~1T(i)   ×[K~1THK~1−K~1THK~2[K~2THK~2]−1K~2THK~1]Δx~1(i)]=∑i=1∞[[Δv~(i)+[K~2THK~2]−1K~2THK~1Δx~1(i)]T   ×(K~2THK~2)[Δv~(i)+[K~2THK~2]−1K~2THK~1Δx~1(i)]   +e~T(i)Q−e~(i)+Δx~1T(i)   ×[K~1THK~1−K~1THK~2[K~2THK~2]−1K~2THK~1]Δx~1(i)].

If we denote $\tilde{Q}={{\tilde{K}}_{1}}^{T}H{\tilde{K}}_{1}-{{\tilde{K}}_{1}}^{T}H{\tilde{K}}_{2}{\left[{\tilde{K}}_{2}^{T}H{\tilde{K}}_{2}\right]}^{-1}{\tilde{K}}_{2}^{T}H{\tilde{K}}_{1}$, it is easy to see $\tilde{Q}\geq 0$.

Let

(39) $$\begin{array}{c} w\left(i\right)=\Delta \tilde{v}\left(i\right)+{\left[{\tilde{K}}_{2}^{T}H{\tilde{K}}_{2}\right]}^{-1}{\tilde{K}}_{2}^{T}H{\tilde{K}}_{1}\Delta {\tilde{x}}_{1}\left(i\right), \end{array}$$

(40) $$\begin{array}{c} R={\left[{\tilde{K}}_{2}^{T}H{\tilde{K}}_{2}\right]}^{-1}{{\tilde{K}}_{2}}^{T}H{\tilde{K}}_{1}. \end{array}$$

From $\Delta {\tilde{x}}_{1}\left(i\right)=\left[\begin{array}{ccc} 0 & 0 & I_{q} \end{array}\right]X_{R0}\left(i\right)$, (39) can be written as

(41) w(i)=Δv~(i)+RΔx~1(i)=Δv~(i)+R[00Iq]XR0(i)=Δv~(i)+K^1XR0(i),

where ${\hat{K}}_{1}=R\left[\begin{array}{ccc} 0 & 0 & I_{q} \end{array}\right]$.

The performance index (38) can continue to be written as

(42) J=∑i=1∞[wT(i)K~2THK~2w(i)+e~T(i)Q−e~(i)+Δx~1T(i)Q~Δx~1(i)]=∑i=1∞[wT(i)K~2THK~2w(i)   +[XR(i)e~(i)Δx~1(i)]T[0000Q−000Q~][XR(i)e~(i)Δx~1(i)]]=∑i=1∞[wT(i)K~2THK~2w(i)+XR0T(i)[0000Q−000Q~]XR0(i)]=∑i=1∞[XR0T(i)Q^XR0(i)+wT(i)K~2THK~2w(i)],

where $\hat{Q}=\left[\begin{array}{ccc} 0 & 0 & 0\\ 0 & \overset{-}{Q} & 0\\ 0 & 0 & \tilde{Q} \end{array}\right]$.

From (41), we derive

(43) $$\begin{array}{c} \Delta \tilde{v}\left(i\right)=w\left(i\right)-{\hat{K}}_{1}X_{R0}\left(i\right).\; \end{array}$$

Substituting (43) into (34), we get

(44) $$\begin{array}{c} X_{R0}\left(i+1\right)=\left(\Phi_{R0}-G_{R0}{\hat{K}}_{1}\right)X_{R0}\left(i\right)+G_{R0}w\left(i\right),\\ \tilde{e}\left(i\right)=C_{R0}X_{R0}\left(i\right). \end{array}$$

Then, the problem becomes an optimal control problem for a normal system (44) under the performance index (42). According to the results in Duan [17], we immediately get the following.

**Theorem 3**

If $\left(\Phi_{R0}-G_{R0}{\hat{K}}_{1}|G_{R0}\right)$ is stabilizable and $\left({\hat{Q}}^{1/2}|\Phi_{R0}-G_{R0}{\hat{K}}_{1}\right)$ is detectable, the optimal regulator of the system (44) minimizing the performance index (42) is given by

(45) w(i)=−[K~2THK~2+GR0TPGR0]−1 ×GR0TP(ΦR0−GR0K^1)XR0(i),

where *P* is the unique symmetric semipositive definite solution of the algebraic Riccati equation:

(46) P=(ΦR0−GR0K^1)TP(ΦR0−GR0K^1) −(ΦR0−GR0K^1)TPGR0[K~2THK~2+GR0TPGR0]−1 ×GR0TP(ΦR0−GR0K^1)+Q^.

## 6. The Existence Conditions of the Optimal Regulator

We will verify the existence conditions of the optimal regulator for (44).

**Theorem 4**

$\left(\Phi_{R0}-G_{R0}{\hat{K}}_{1}|G_{R0}\right)$ is stabilizable if and only if (Φ_*R*0_ | *G*_*R*0_) is stabilizable.

**Proof**

Notice that the system (44) is derived from the system (34) under the state feedback (43). We know that the state feedback does not change the stabilizability of the system as discussed by [17], so the system (44) is stabilizable if and only if the system (34) is stabilizable; that is, $\left(\Phi_{R0}-G_{R0}{\hat{K}}_{1}|G_{R0}\right)$ is stabilizable if and only if (Φ_*R*0_ | *G*_*R*0_) is stabilizable. This completes the proof.

**Theorem 5**

(Φ_*R*0_ | *G*_*R*0_) is stabilizable if and only if $\left({\overset{-}{A}}_{1}^{N}|{\tilde{B}}_{1}\right)$ is stabilizable and

(47) $$\begin{array}{c} \left[\begin{array}{cc} {\overset{-}{A}}_{1}^{N}-I & {\tilde{B}}_{1}\\ {\tilde{C}}_{1} & {\tilde{C}}_{2} \end{array}\right] \end{array}$$

is of full row rank.

**Proof**

First, we have

(48) rank⁡[λI−ΦR0GR0]=rank⁡[λI−AR00−GPRλI−ΦG]=mS+rank⁡[λI−ΦG].

Noticing the structure of Φ and *G*, Theorem 5 can be proved by Lemma 1(a) in Liao et al. [14]. Here we omit the proof.

Note that the matrix in (47) is Ψ in (A7).

**Theorem 6**

$\left(\begin{array}{cc} {\overset{-}{A}}_{1}^{N} & {\tilde{B}}_{1} \end{array}\right)$ is stabilizable if and only if (A1) holds.

**Proof**

First, from [8], we know that $\left(\begin{array}{cc} {\overset{-}{A}}_{1}^{N} & {\tilde{B}}_{1} \end{array}\right)$ is stabilizable if and only if the system (7) is stabilizable.

By using formula (6) and the nonsingularity of $\left[\begin{array}{cc} P_{1} & 0\\ 0 & I \end{array}\right]$ and *Q*_1_, we have

(49) rank⁡[λE−(A+BK)B]  =rank⁡(Q1[λE−(A+BK)B][P100I])  =rank⁡[λQ1EP1−Q1(A+BK)P1Q1B]  =rank⁡[λ[I000]−[A11A12A21A22][B1B2]].

So, the system (7) is stabilizable if and only if the system (4) is stabilizable.

By $\mathrm{rank}\left[\begin{array}{cc} \lambda E-\left(A+BK\right) & B \end{array}\right]$ = $\mathrm{rank}\left[\begin{array}{cc} \lambda E-A & B \end{array}\right]\left[\begin{array}{cc} I & 0\\ -K & I \end{array}\right]$ = $\mathrm{rank}\left[\begin{array}{cc} \lambda E-A & B \end{array}\right]$, the system (4) is stabilizable if and only if the system (1) is stabilizable; that is, (A1) holds.

In summary, this completes the proof.

**Remark 7**

This theorem also proves that the systems (7) and (1) have the same stabilizability.

Combining Theorems 4, 5, and 6, if the original system (1) is stabilizable and Ψ in (A7) is of full row rank, the final formal system (44) is also stabilizable. Furthermore, the condition is both necessary and sufficient. These conditions ensure that the state feedback gain in Theorem 3 exists.

Next, we examine the detectability of $\left({\hat{Q}}^{1/2}|\Phi_{R0}-G_{R0}{\hat{K}}_{1}\right)$.

**Theorem 8**

If (A2) holds, the system (4) is detectable.

**Proof**

Since the output feedback does not change the detectability of the system as discussed by [2], this completes the proof.

**Theorem 9**

The system (4) is detectable if and only if $\left(\begin{array}{cc} {\overset{-}{C}}_{1} & {\overset{-}{A}}_{1} \end{array}\right)$ is detectable.

**Proof**

First, by the Popov-Belevitch-Hautus (PBH) rank test as discussed by [17], the system (4) is detectable if and only if, for any complex *λ* satisfying |*λ*| ≥ 1,

(50) $$\begin{array}{c} \mathrm{rank}\left[\begin{array}{c} \lambda E-\left(A+BK\right)\\ C \end{array}\right]=n\left(\text{full}\;\text{column}\;\mathrm{rank}\right). \end{array}$$

By using formula (6) and the nonsingularity of $\left[\begin{array}{cc} Q_{1} & 0\\ 0 & I \end{array}\right]$ and *P*_1_, we have

(51) rank⁡[λE−(A+BK)C]  =rank⁡([Q100I][λE−(A+BK)C]P1)  =rank⁡[λQ1EP−Q1(A+BK)P1CP1]  =rank⁡[λ[I000]−[A11A12A21A22][C1C2]].

This shows that the systems (4) and (7) have the same detectability.

Again from [8], the system (7) is detectable if and only if $\left(\begin{array}{cc} {\overset{-}{C}}_{1} & {\overset{-}{A}}_{1} \end{array}\right)$ is detectable.

In summary, this completes the proof.

**Theorem 10**

$\left(\begin{array}{cc} {\overset{-}{C}}_{1} & {\overset{-}{A}}_{1} \end{array}\right)$ is detectable if and only if $\left(\begin{array}{cc} {\tilde{C}}_{1} & {\overset{-}{A}}_{1}^{N} \end{array}\right)$ is detectable.

This theorem is a proven lemma in [8, 14].

**Theorem 11**

If $\left(\begin{array}{cc} {\tilde{C}}_{1} & {\overset{-}{A}}_{1}^{N} \end{array}\right)$ is detectable, $\left({\hat{Q}}^{1/2}|\Phi_{R0}-G_{R0}{\hat{K}}_{1}\right)$ is detectable.

**Proof**

First, we have

(52) rank⁡[λI−(ΦR0−GR0K^1)Q^1/2]  =rank⁡[λI−AR00Im(λ−1)Im−C~1+C~2R00λI−A−1N+B~1R0000Q−1/2000Q~1/2]  =rank⁡[−C~1+C~2RλI−A−1N+B~1RQ~1/2].

Assuming $V=\left[\begin{array}{c} H^{1/2}{\tilde{K}}_{1}\\ j{\left\{{\left[H{\tilde{K}}_{2}\right]}^{-1}\right\}}^{1/2}{\tilde{K}}_{2}^{T}H{\tilde{K}}_{1} \end{array}\right]$, we have $\tilde{Q}=V^{T}V$. So

(53) rank⁡[−C~1+C~2RλI−A−1N+B~1RQ~1/2]  =rank⁡[−C~1+C~2RλI−A−1N+B~1RV]  =rank⁡[−C~1+C~2RλI−A−1N+B~1RH1/2K~1j{[K~2THK~2]−1}1/2K~2THK~1]  =rank⁡[−C~1λI−A−1NK~10]=rank⁡[λI−A−1NC~1K~1].

If $\left(\begin{array}{cc} {\tilde{C}}_{1} & {\overset{-}{A}}_{1}^{N} \end{array}\right)$ is detectable, $\left[\begin{array}{c} \lambda I-{\overset{-}{A}}_{1}^{N}\\ {\tilde{C}}_{1}\\ {\tilde{K}}_{1} \end{array}\right]$ is of full column rank.

This completes the proof.

Combining Theorems 8 and 11, if the original system (1) is detectable, $\left({\hat{Q}}^{1/2}|\Phi_{R0}-G_{R0}{\hat{K}}_{1}\right)$ is also detectable. Furthermore, the condition is just sufficient.

## 7. The Optimal Preview Controller for the Original System

Returning to the optimal control input (45) of the descriptor augmented error system and the related formula (43), we get

(54) Δv~(i) =w(i)−K^1XR0(i) =−[K~2THK~2+GR0TPGR0]−1  ×GR0TP(ΦR0−GR0K^1)XR0(i)−K^1XR0(i) ={−[K~2THK~2+GR0TPGR0]−1GR0TP(ΦR0−GR0K^1)−K^1}  ×XR0(i)=TXR0(i),

where $T=-{\left[{\tilde{K}}_{2}^{T}H{\tilde{K}}_{2}+G_{R0}^{T}PG_{R0}\right]}^{-1}G_{R0}^{T}P\left(\Phi_{R0}-G_{R0}{\hat{K}}_{1}\right)-{\hat{K}}_{1}$.

From (21) and (54), we continue to get

(55) Δu~(i)=K~1Δx~1(i)+K~2Δv~(i)=K~1Δx~1(i)+K~2TXR0(i)=K~1[00Iq]XR0(i)+K~2TXR0(i)={K~1[00Iq]+K~2T}XR0(i)={K~1[00Iq]+K~2T}[XR(i)e~(i)Δx~1(i)]=T^[XR(i)e~(i)Δx~1(i)],

where $\hat{T}={\tilde{K}}_{1}\left[\begin{array}{ccc} 0 & 0 & I_{q} \end{array}\right]+{\tilde{K}}_{2}T$.

Noticing

(56) $$\begin{array}{c} X_{R}\left(i\right)=\left[\begin{array}{c} \Delta \tilde{R}\left(i\right)\\ \Delta \tilde{R}\left(i+1\right)\\ \vdots \\ \Delta \tilde{R}\left(i+S-1\right) \end{array}\right], \end{array}$$

$\hat{T}$ is partitioned into

(57) $$\begin{array}{c} \hat{T}=\left[\begin{array}{cccc} T_{R}\left(0\right) & T_{R}\left(1\right) & \cdots & T_{R}\left(S-1\right)\;|\;T_{e}\;|\;T_{x} \end{array}\right]. \end{array}$$

Equation (55) can be written as

(58) $$\begin{array}{c} \Delta \tilde{u}\left(i\right)=\sum_{l=0}^{S-1}T_{R}\left(l\right)\Delta \tilde{R}\left(i+l\right)+T_{e}\tilde{e}\left(i\right)+T_{x}\Delta {\tilde{x}}_{1}\left(i\right). \end{array}$$

Noticing

(59) $$\begin{array}{c} \Delta \tilde{u}\left(i\right)=\left[\begin{array}{c} \Delta u\left(iN\right)\\ \Delta u\left(iN+1\right)\\ \vdots \\ \Delta u\left(iN+N-1\right) \end{array}\right], \end{array}$$

*T*_*R*_(*l*), *T*_*e*_, and *T*_*x*_ are decomposed into

(60) TR(l)=[TR(0)(l)TR(1)(l)⋮TR(N−1)(l)], Te=[Te(0)Te(1)⋮Te(N−1)], Tx=[Tx(0)Tx(1)⋮Tx(N−1)]                      (l=0,1,…,S−1).

Then (58) can be written as

(61) [Δu(iN)Δu(iN+1)⋮Δu(iN+N−1)]=∑l=0S−1[TR(0)(l)TR(1)(l)⋮TR(N−1)(l)]ΔR~(i+l)+[Te(0)Te(1)⋮Te(N−1)]e~(i)+[Tx(0)Tx(1)⋮Tx(N−1)]Δx~1(i).

The above equation can be further written as

(62) $$\begin{array}{c} \Delta u\left(iN+j\right)=\sum_{l=0}^{S-1}T_{R}^{\left(j\right)}\left(l\right)\Delta \tilde{R}\left(i+l\right)+T_{e}^{\left(j\right)}\tilde{e}\left(i\right)+T_{x}^{\left(j\right)}\Delta {\tilde{x}}_{1}\left(i\right). \end{array}$$

That is,

(63) u((i+1)N+j)=u(iN+j)+∑l=0S−1TR(j)(l)ΔR~(i+l)+Te(j)e~(i)+Tx(j)Δx~1(i).

If *i* is substituted by *i* − 1, we obtain the control input of the most important theorem.

**Theorem 12**

If (A1)–(A7) hold and *Q*_*e*_ > 0 and *H*_*u*_ > 0, then the Riccati equation (46) has a unique symmetric semipositive definite solution, and the optimal control input of the system (1) is

(64) u(iN+j)=u((i−1)N+j)+∑l=0S−1TR(j)(l)ΔR~(i+l−1)+Te(j)e~(i−1)+Tx(j)[x1(iN)−x1((i−1)N)]       i=1,2,…;  j=0,1,2,…,N−1,

where

(65) $$\begin{array}{c} \Delta \tilde{R}\left(i-1\right)=\left[\begin{array}{c} R\left(iN\right)-R\left(\left(i-1\right)N\right)\\ R\left(iN+1\right)-R\left(\left(i-1\right)N+1\right)\\ \vdots \\ R\left(iN+N-2\right)-R\left(\left(i-1\right)N+N-2\right)\\ R\left(iN+N-1\right)-R\left(\left(i-1\right)N+N-1\right) \end{array}\right],\\ \tilde{e}\left(i-1\right)=\left[\begin{array}{c} e\left(\left(i-1\right)N\right)\\ e\left(\left(i-1\right)N+1\right)\\ \vdots \\ e\left(\left(i-1\right)N+N-2\right)\\ e\left(\left(i-1\right)N+N-1\right) \end{array}\right] \end{array}$$

are determined by

(66) $$\begin{array}{c} \tilde{e}\left(i-1\right)={\tilde{C}}_{1}{\tilde{x}}_{1}\left(i-1\right)-\tilde{R}\left(i-1\right)+{\tilde{C}}_{2}\tilde{v}\left(i-1\right), \end{array}$$

where

(67) $$\begin{array}{c} {\tilde{x}}_{1}\left(i-1\right)=x_{1}\left(\left(i-1\right)N\right),\\ \tilde{R}\left(i-1\right)=\left[\begin{array}{c} R\left(\left(i-1\right)N\right)\\ R\left(\left(i-1\right)N+1\right)\\ \vdots \\ R\left(\left(i-1\right)N+N-2\right)\\ R\left(\left(i-1\right)N+N-1\right) \end{array}\right]. \end{array}$$

In addition, $\tilde{v}\left(i-1\right)$ can be derived from (21) and (A6) as follows:

(68) $$\begin{array}{c} \tilde{v}\left(i-1\right)={\tilde{K}}_{2}^{-1}\tilde{u}\left(i-1\right)-{\tilde{K}}_{2}^{-1}{\tilde{K}}_{1}{\tilde{x}}_{1}\left(i-1\right), \end{array}$$

where

(69) $$\begin{array}{c} \tilde{u}\left(i-1\right)=\left[\begin{array}{c} u\left(\left(i-1\right)N\right)\\ u\left(\left(i-1\right)N+1\right)\\ \vdots \\ u\left(\left(i-1\right)N+N-2\right)\\ u\left(\left(i-1\right)N+N-1\right) \end{array}\right]. \end{array}$$

## 8. Numerical Example

Consider the following regular linear discrete-time descriptor noncausal system in the form of (1):

(70) $$\begin{array}{c} \left[\begin{array}{ccc} 1 & 0 & 0\\ 0 & 0 & 1\\ 0 & 0 & 0 \end{array}\right]x\left(k+1\right)=\left[\begin{array}{ccc} 0 & 1 & 0\\ 1 & -2 & 2\\ 0 & 0 & -1 \end{array}\right]x\left(k\right)+\left[\begin{array}{c} 1\\ 0\\ 1 \end{array}\right]u\left(k\right),\\ y\left(k\right)=\left[\begin{array}{ccc} 1 & -1 & 1 \end{array}\right]x\left(k\right). \end{array}$$

In this case, the coefficient matrices are

(71) $$\begin{array}{c} E=\left[\begin{array}{ccc} 1 & 0 & 0\\ 0 & 0 & 1\\ 0 & 0 & 0 \end{array}\right],\;\;A=\left[\begin{array}{ccc} 0 & 1 & 0\\ 1 & -2 & 2\\ 0 & 0 & -1 \end{array}\right],\\ B=\left[\begin{array}{c} 1\\ 0\\ 1 \end{array}\right],\;\;C=\left[\begin{array}{ccc} 1 & 0 & 1 \end{array}\right], \end{array}$$

respectively.

Through calculating, the above system satisfies all conditions required in the paper. By MATLAB simulation, the gain matrix in output feedback is taken as *M* = 2, and the coefficient matrices in (7) are

(72) $$\begin{array}{c} A_{11}=\left[\begin{array}{cc} 2 & -1\\ 1 & -2 \end{array}\right],\;\;A_{12}=\left[\begin{array}{c} 3\\ 4 \end{array}\right],\;\;A_{21}=\left[\begin{array}{cc} 2 & -2 \end{array}\right],\\ A_{22}=3,\;\;B_{1}=\left[\begin{array}{c} 1\\ 0 \end{array}\right],\;\;B_{2}=1,\\ C_{1}=\left[\begin{array}{cc} 1 & -1 \end{array}\right],\;\;C_{2}=2. \end{array}$$

We assume that *N* = 3 in (A4). To calculate ${\overset{-}{A}}_{1}$, ${\overset{-}{B}}_{1}$, ${\overset{-}{C}}_{1}$, ${\overset{-}{C}}_{2}$, and Ψ give

(73) A−1=[01−1.66670.6667],  B−1=[0−1.3333],C−1=[−0.33330.3333],  C−2=−0.6667,Ψ=[A−13−IA−12B−1A−1B−1B−1C−1C−200C−1A−1C−1B−1C−20C−1A−12C−1A−1B−1C−1B−1C−2]=[−2.1111−1.2222−0.8889−1.333302.0370−2.92591.6296−0.8889−1.3333−0.33330.3333−0.666700−0.5556−0.1111−0.4444−0.666700.1852−0.62960.1481−0.4444−0.6667].  

Let the initial state vector ${\tilde{x}}_{1}\left(0\right)=\left[\begin{array}{c} -2\\ -1 \end{array}\right]$. In addition, take the weight matrices *Q*_*e*_ = 100 and *H*_*u*_ = 10. Let preview length be *M*_*R*_ = 30; that is, *S* = 10. We present MATLAB simulation results for two cases.

*(1)  Step Function.* Let the desired signal be

(74) $$\begin{array}{c} R\left(k\right)=\{\begin{array}{cc} 0, & k\leq 50\\ 5, & k>50. \end{array} \end{array}$$

**Figure 1:**
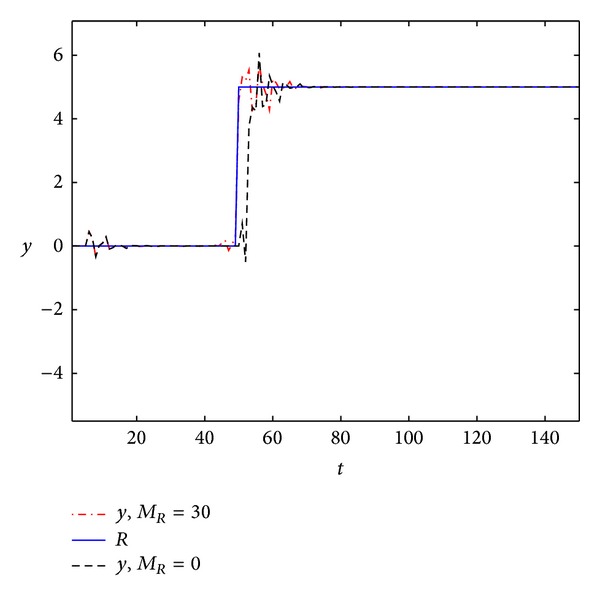
The output response to step function.

**Figure 2:**
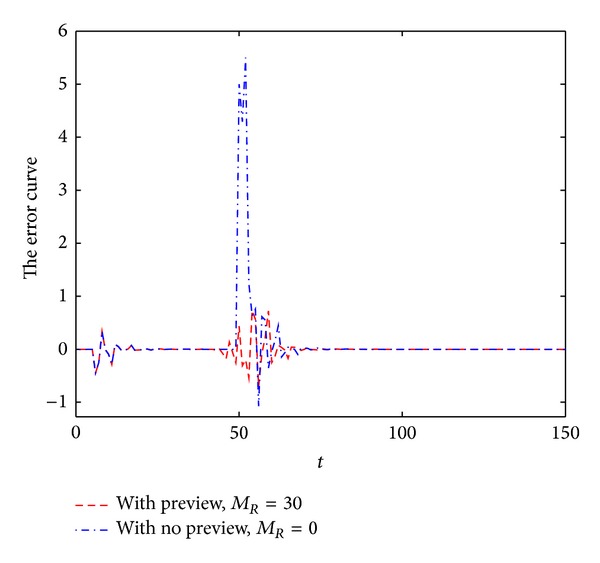
The error of step function.

By MATLAB simulation, the output response of the linear discrete-time descriptor noncausal multirate system (with preview action and no preview action) is shown in Figure 1. The error signals are shown in Figure 2. Note that the preview action significantly reduces the error. In particular, the error signal is asymptotically zero.

*(2)  Ramp Function*. Let the desired signal be

(75) $$\begin{array}{c} R\left(k\right)=\{\begin{array}{cc} 0, & k\leq 30\\ 0.25\left(k-30\right), & 30<k\leq 50\\ 5, & k>50. \end{array} \end{array}$$

**Figure 3:**
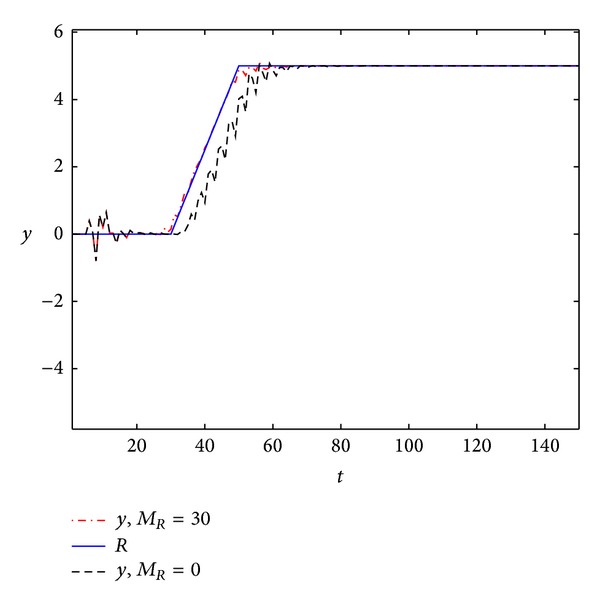
The output response to ramp function.

**Figure 4:**
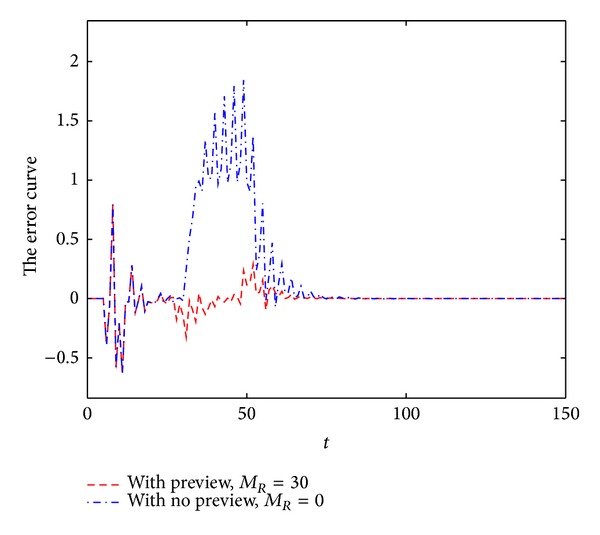
The error of ramp function.

The output responses are shown in Figure 3. The error signals are shown in Figure 4.

From Figures 1–4, we can easily see the effectiveness of the present controller of this paper. On the one hand, when using preview control, the output curve can track the desired signal faster; on the other hand, the overshoot is smaller.

## 9. Conclusion

This paper studied the optimal preview controller for linear discrete-time descriptor noncausal multirate systems. By making use of the characteristics of causal controllability and causal observability, the original system was converted into a descriptor causal closed-loop system. Then, using the characteristics of a causal system and a discrete lifting technique, the descriptor causal closed-loop multirate system was changed into a single-rate normal system. Taking advantage of the conventional method of the error system in preview control theory, a descriptor augmented error system is constructed, and the problem is transformed into a regulator problem. Finally, the optimal preview controller is designed according to the related theory of preview control. From preview control theory, the obtained closed-loop system contains an integrator so that the response of the system does not have static error. The numerical simulation showed the effectiveness of the proposed preview control system.
